# Prospective Application of Activity-Based Proteomic Profiling in Vision Research-Potential Unique Insights into Ocular Protease Biology and Pathology

**DOI:** 10.3390/ijms20163855

**Published:** 2019-08-08

**Authors:** Hui Peng, John D. Hulleman

**Affiliations:** 1 Department of Ophthalmology, University of Texas Southwestern Medical Center, 5323 Harry Hines Blvd, Dallas, TX 75390-9057, USA; 2 Department of Pharmacology, University of Texas Southwestern Medical Center, 5323 Harry Hines Blvd, Dallas, TX 75390, USA

**Keywords:** proteases, enzyme activity, chemical biology, age-related macular degeneration, retinal degeneration, eye disease

## Abstract

Activity-based proteomic profiling (ABPP) is a powerful tool to specifically target and measure the activity of a family of enzymes with the same function and reactivity, which provides a significant advantage over conventional proteomic strategies that simply provide abundance information. A number of inherited and age-related eye diseases are caused by polymorphisms/mutations or abnormal expression of proteases including serine proteases, cysteine proteases, and matrix metalloproteinases, amongst others. However, neither conventional genomic, transcriptomic, nor traditional proteomic profiling directly interrogate protease activities. Thus, leveraging ABPP to probe the activity of these enzyme classes as they relate to normal function and pathophysiology of the eye represents a unique potential opportunity for disease interrogation and possibly intervention.

## 1. Introduction

Whereas the terms “genome” and “transcriptome” refer to the entire cellular DNA and RNA content, respectively, the term, “proteome”, was first used nearly 25 years ago to describe the group of proteins that are expressed by the genome, along with “proteomics”, which refers to the study of the proteome [1]. So far, entire genomes from numerous organisms have been fully sequenced, and the proteomes of various organisms ranging from yeast [2,3] to mammals (mouse [4], human [5]), have been mapped. This wealth of information enables a near comprehensive understanding and annotation of complex biological systems and processes.

Data-rich techniques such as global transcriptomic analysis [6] have provided diagnostic insight into potential genes and pathways that are responsive to genetic/environmental stresses and are involved in disease. However, the proteome, the ultimate product of genomic information, is inherently more complex than the corresponding transcriptional information would predict due to the potential for additional posttranscriptional (e.g., alternative splicing) and post-translational modifications (PTMs, e.g., oxidation, phosphorylation, acetylation, etc.). Therefore, to better understand cellular and organismal function under normal and pathologic conditions, proteomics has been broadened to include site-specific PTMs, comparison of protein levels under different conditions, and the study of protein-protein interactions [7]. Advances in liquid chromatography-mass spectrometry/mass spectrometry (LC-MS/MS) have allowed for effective identification and quantitation of proteins with relative ease [8,9]. Nonetheless, some intrinsic properties of traditional MS-based proteomic profiling limit the assessment of protein activities associated with organism function or biomarker discovery. For example, due to the complexity of samples, the sensitivity limit of MS instruments, and differential peptide ionization/fragmentation, the detection of proteins is biased towards highly abundance peptides. These phenomena make biologically active, but low abundance proteins difficult to detect [10,11]. Moreover, traditional proteomics only compare protein abundance; however, proteases, which play key roles in both normal physiological and pathological processes, such as inflammation [12] and angiogenesis [13], do not necessarily increase in abundance when activated, but instead their activities are regulated by inhibitor binding, propeptide cleavage, or PTMs [14].

**Figure 1:**
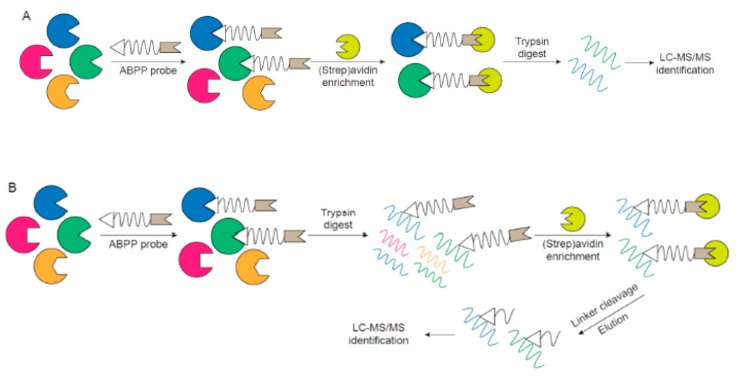
Overview of activity-based proteomic profiling (ABPP) followed by SDS-PAGE analysis. (**A**) General design of an ABPP probe containing a reactive group, a linker region and a fluorescent or affinity tag. (**B**) Example reaction of a fluorescent ABPP probe with a sample containing enzymes followed by SDS-PAGE separation and detection of labeled proteins by fluorescence gel scanning.

**Figure 2:**
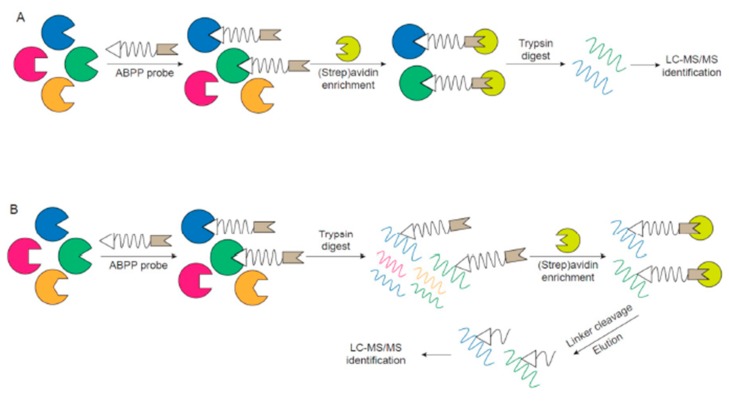
Example workflow of ABPP probe labeling followed by liquid chromatography-mass spectrometry/mass spectrometry (LC-MS/MS) analysis. (**A**) Representative scheme of ABPP probe labeling of samples proceeded by enrichment (i.e., avidin isolation of biotin-tagged probes), trypsinization and LC-MS/MS identification. (**B**) An alternative LC-MS/MS approach to directly identify the active site labeling by the ABPP probe using a cleavable linker sequence.

Given the inability of traditional transcriptomic and proteomic strategies to accurately probe protein activities as they relate to diseases and biological functions, activity-based proteomic profiling (ABPP) is an attractive complementary or standalone technique for assessing protein activities and exploring functional proteomics. ABPP involves using an active-site-directed fluorescent or affinity-tagged probe to covalently bind to a specific group of enzymes within the total proteome (Figure 1A,B). Since this process mimics the catalytic reaction, only the active enzymes can react with ABPP probes. After labeling, the modified enzymes can be visualized by fluorescent imaging of SDS-PAGE gels (Figure 1B) or enriched through affinity purification followed by LC-MS/MS for detection (Figure 2A,B). Consequently, depending on the chemical probes utilized, only proteins with specific activities, which are likely to be the key players in disease pathogenesis, are visualized/identified. ABPP has been applied to study various diseases, such as cancer [15,16,17], neurodegeneration [18], microbial infection [19,20], biomarker discovery, inhibitor screens, and drug development.

Although optical imaging (e.g., fundus imaging, optical coherence tomography, quantitative autofluorescence, etc.) and therapeutics (i.e., anti-vascular endothelial growth factor antibodies and adeno-associated virus gene replacement) have been rapidly developed and applied to eye research, due to the complex anatomy and physiology of the eye, the molecular mechanisms of many eye diseases are still poorly understood. Proteomics can serve as an applicable tool to study the pathogenesis, diagnosis, and treatment of eye diseases [21]. A number of publications have reported the proteomes detected in different parts of the eye, such as aqueous humor [22], cornea [23,24], lens [25], vitreous body [26,27], retina [27] and retinal pigment epithelium (RPE)/choroid [28], under healthy and diseased conditions, and have observed that a number of proteases may be linked to eye diseases. Alternatively, genome-wide association studies (GWASs) have also been conducted to identify risk-associated genes and mutations that correlate with eye diseases. Interestingly, many of the disease-associated genes encode for proteases [29]. However, a comprehensive study of actual protease activities in normal and pathologic conditions of the eye is still lacking. To bridge this gap in knowledge, ABPP, which has been virtually unutilized in eye research, is poised to provide insights into ocular biology and serves as the missing functional link in omics-centered research.

In this review, we will focus on potential applications of ABPP to study the pathology of eye diseases from the perspective of ABPP experimental design, the involvement of proteases in eye disease pathogenesis, and how ABPP can be employed for disease biomarker identification, disease diagnosis and drug discovery.

## 2. ABPP Experimental Design

At the heart of an ABPP experiment is a probe that specifically binds to the active site of a protein and an analytical analysis platform that identifies and quantifies probed proteins or peptides. The probe can be divided into three fundamental building blocks, a reactive group that can covalently bind to the active site of a specific subset of proteins, a linker region or binding group that enhances the selectivity toward desired proteins, and a reporter tag that enables the probed proteins to be visualized or isolated and enriched for further identification (Figure 1A) [14,30].

Depending on the catalytic mechanism of different categories of proteases, a reactive group can be designed accordingly. To be more specific, protease serine, cysteine and threonine proteases involve nucleophilic attack directly by the hydroxyl or thiol group in the active site of the protease with the formation of an acyl–enzyme intermediate. Therefore, electrophiles can be incorporated as the reactive group in the probe for the profiling of such proteases. For example, the electrophile, fluorophosphonate, which specifically and irreversibly inhibits serine proteases, is used in the design of most serine protease ABPP probes [31]. In contrast to enzyme-bound nucleophiles, aspartyl proteases and metalloproteases employ a metal-activated water molecule for catalysis, which makes the use of an electrophile ABPP probe not feasible in these instances. Consequently, affinity-based reactive groups have been synthesized, such as reversible inhibitors, conjugated with photocrosslinkers (e.g., benzophenone, diazirine), which, upon UV light exposure, generate radicals that covalently bind to proteins on spatially close atoms [32,33,34]. 

The linker separates the reactive group from the reporter tag, which reduces steric hindrance and improves the accessibility of the reactive group to the active site of protein. Besides using extended alkyl or polyethylene glycol spacers, the linker can also be designed to enhance the specificity of the ABPP probe by incorporating the substrate sequence for a better recognition [35]. Research groups often use probes with a short peptide linker, in which the sequence mimics the optimal substrate of proteases, and this short peptide sequence can even be specialized to probe subfamilies of proteases [36]. Alternatively, enzymatically or chemically cleavable linkers (e.g., tobacco etch virus (TEV) protease recognition sequence [37], diazobenzenes [38]) have also been developed, which prevent nonspecifically bound peptides from being eluted along with properly probed proteins during the enrichment process, thus allowing a cleaner background for detection. In addition, peptides labeled with ABPP probes of lower molecular weight can be more efficiently ionized and enable the identification of the active site probed in the protease (Figure 2).

The purpose of the reporter tag is to isolate, enrich and/or detect active proteins, thus selection of the reporter tag is associated with the choice of analytical analysis platform. Fluorescent or radioactive tags are often used for gel-based methods, where the targets in healthy vs. diseased samples, for example, can be easily separated, visualized, and compared rapidly (Figure 1B). While the advantage of a gel-based platform is the robustness, relative high-throughput, and a quick parallel comparison, the major limitation of this strategy is lack of resolution and identification of protein targets simply by apparent molecular weight. Accordingly, groups have synthesized ABPP probes with affinity tags, such as biotin, which can be efficiently isolated and enriched using an avidin resin. There are two different ways to perform ABPP with an affinity tag (Figure 2A,B). One way is the so-called ABPP and multidimensional protein identification technology (ABPP-MudPIT), which involves the labeling of active proteins with biotinylated probe, incubation with avidin resin, tryptic digestion of enriched proteins and MS identification of peptides (Figure 2A). However, the active site information is often lost since the peptides labeled with the probe are still bound to the resin. Another method is to digest the labeled proteins into peptides, incubate peptides with the resin and elute the labeled peptides from the resin by employing cleavable linkers for final identification by MS (Figure 2B). This second method can provide active site information of the probe and further confirm that the ABPP probe is selective and only targets the active site of desired proteins.

## 3. Proteases in Eye Disease

Researchers have developed ABPP probes for various protein classes with active sites, including proteases, which have been shown to contribute to numerous biological processes, such as protein turnover, processing of cellular information, generation and transduction of biological signals, and influencing DNA replication and transcription, thus being critically important in the development of many diseases [39] (e.g., cardiovascular disease [40], Alzheimer disease [41], inflammatory disease [42]). In this regard, a wide range of proteases are also critically important in maintaining normal ocular physiology and are likely involved in disease-related processes. Next, we summarize representative groups of proteases that have been studied the most through genetic analysis or GWAS and identified as being important in maintaining eye health. These proteases may be functionally altered in eye diseases, and are thus of specific interest to target and evaluate with ABPP.

### 3.1. Serine Proteases

The pathology of age-related macular degeneration (AMD) is suggested to be influenced by polymorphisms and/or substantially altered expression levels of complement factors, including complement factor B (CFB) and complement factor I (CFI), both of which are serine proteases [43,44]. CFB is a component of the alternative complement activation pathway, and upon cleavage by complement factor D (CFD), the catalytic subunit of CFB, Bb, is generated. This subunit has serine protease activity and yields the formation of the C3 convertase complex by binding to C3b [45]. Via single nucleotide polymorphism (SNP) and haplotype analyses related to AMD, it has been shown that variants in *CFB* correlate with AMD pathogenesis independently, or in combination with other gene variants. Specifically, a variant of CFB, R32Q (rs641153), was reported to significantly reduce the risk of AMD, most likely by decreasing the binding affinity of CFB to C3b [46]. The R32Q variant in CFB was also found to be protective along with the variant of intron 10 of *C2* (rs547154) [43], as well as the R102G variant of C3 (rs2230199) and the V62I variant of CFH (rs800292) [47]. However, evidence that patients with R32Q CFB have lower AMD risk is not fully convincing [48]. Besides sequencing the CFB coding sequence to identify genetic alterations, the expression levels of CFB have been quantified both in circulation and in the human eye. CFB is elevated in blood samples of AMD patients at both the transcript and protein level [49,50] and CFB protein expression is increased proportionally with the severity of AMD in the vitreous and Bruch’s membrane-choroid complex [51].

Similar to CFB, genetic variations in CFI associated with AMD have also been identified. CFI is a serine protease which is usually maintained in an inactive zymogen form, and upon activation by furin, CFI terminates the complement pathway by cleaving cell-bound or fluid phase C3b and C4b [52]. GWAS or sequencing of *CFI* exons in AMD patients revealed that several rare mutations located near the catalytic chain of CFI (G119R [53,54,55,56,57,58] (rs141853578), G188A [53] (rs774141891)) were correlated with AMD development. The impact of these CFI variants was further characterized, revealing that patients carrying these mutations often had lower expression levels of CFI in the serum, which consequently decreased the regulatory capacity of CFI to inhibit complement pathways [56,58]. Another study supported this observation by showing that CFI G119R and G188A resulted in reduced CFI levels in plasma, and recombinant mutant CFI is expressed at lower levels and inefficiently secreted compared to wild-type CFI in human cells [53]. Moreover, CFD is also a serine protease, but the findings of whether CFD SNPs correlate to AMD development are not consistent [59,60].

In addition to complement factors, an SNP (rs11200638) in the promoter region, as well as two synonymous SNPs (rs2293870 G>C or T, rs1049331 C>T), in high temperature requirement protein A1 (*HTRA1*), which encodes a secreted serine protease, have also been confirmed as major genetic risk factors of AMD and estimated to present a population attributable risk of ~50% [61,62,63]. HTRA protein families are ubiquitously expressed in mammalian tissues and are involved in various important biological processes, including protein quality control that is critical to cell fate [64]. HTRA proteins also play significant roles in the pathogenesis of diseases such as cancer, neurodegenerative disorders, and arthritic diseases [65]. The risk alleles are linked to the elevated expression of *HTRA1* at both the transcript and protein levels in lymphocytes and RPE cells of AMD patients, and HTRA1 expression co-localized with drusen [62] and AMD lesions [66]. This observation was supported by a proteomic comparison that showed that the RPE cells derived from AMD patients with an *HTRA1* variant (rs11200638) have higher levels of HTRA1 than control cells [67]. Elevated expression levels of HTRA1 have been shown to associate with the development of eye diseases. For example, overexpression of human HTRA1 in mice RPE generated fundamental features of polypoidal choroidal vasculopathy, exhibited higher risk for choroidal neovascularization (CNV) and displayed RPE atrophy and photoreceptor degeneration [68]. In another animal model for eye research, zebrafish, RPE cells as well as photoreceptors were also observed to have increased HTRA1 levels when carrying the rs11200638 variant of *HTRA1* [69]. Interestingly, photoreceptor-specific overexpression of HTRA1 in zebrafish induced photoreceptor death and changes in RPE morphology, including lipofuscin accumulation [69].

Although genetic variations have been identified in serine proteases and in some cases, protein expression levels have been quantified, the activities of these serine proteases have not been targeted directly and compared. Serine protease activity is not only dependent on the abundance of the protease, but also on the absence or presence of endogenous serine protease inhibitors, or serpins, which have been associated with a variety of angiogenesis-related eye diseases (e.g., pigment epithelium derived factor, PEDF) [70], detected in ocular-derived cell lines (e.g., serine protease inhibitor E1, SERPINE1 [a.k.a. heat-shock protein 47, HSP47]) [71], as well as in human tear fluid (e.g., secretory leukocyte protease inhibitor) [72]. Therefore, how the activities of these serine proteases are modulated and play a role in AMD and other eye diseases is still unknown. The connections between HTRA1 protein activities and their impact on AMD development especially needs further investigation, and it is feasible that ABPP probes would be able to contribute in this regard.

### 3.2. Cysteine Proteases

Caspases are cysteine-aspartic proteases involved in mediating key decisions involving programmed cell death and inflammation, which use a nucleophilic cysteine in the enzyme active site to attack an amide bond of substrate proteins, resulting in peptide bond cleavage at an aspartic acid residue [73,74]. Caspases are expressed as inactive zymogens and assemble into dimers or macromolecular complexes to obtain catalytic activities upon appropriate biological signals [75]. Retinal ganglion cell (RGC) death, which usually takes place after ocular injury or during degenerative diseases of the eye (e.g., ischemic optic neuropathy, glaucoma, retinal detachment and diabetic retinopathy), is reported to be caspase-dependent, involving the activation of caspase-2, caspase-3, caspase-6, caspase-7, caspase-8 and/or caspase-14 [76,77,78,79,80,81,82]. Caspase-dependent RGC loss has been tested in various ocular injury and disease models, revealing elevated levels of cleaved caspases in RGCs by Western blotting and immunohistochemistry. Furthermore, knockdown or inhibition of caspases can partially restore RGCs and vision function that are reduced in disease models [77,79,83,84]. Besides accelerating RGC death induced by ocular disease, caspase-14 can also modulate RPE cell barrier functions under hyperglycemia conditions, including compromising transcellular electrical resistance [85]. In addition to the regulation of RGC death by the aforementioned caspases, other caspases including caspase-1 and caspase-4 are involved in inflammasome activation, which has been suggested as a pathologic feature of dry AMD and RPE degeneration [86], and are gene therapies targets to treat inflammatory diseases, such as uveitis [87].

Calpains are ubiquitous, calcium-dependent, non-lysosomal cysteine proteases that, like caspases, can also regulate apoptosis [88]. Although the function of calpains is not fully understood, they have been implicated in multiple calcium-regulated physiological processes, such as cytoskeletal rearrangement and cell proliferation in a broad range of organs including brain, skeletal muscle and eye [89,90]. Elevated levels of activated calpain have been correlated with glaucoma progression. In a rat experimental model of glaucoma, calpain cleavage and the hydrolyzed substrate were detected in RGCs by immunoblot analysis under conditions of increased intraocular pressure [91]. Activated calpain 1 and calpain 2 were also detected in a monkey model of retinal hypoxic damage, and an inhibitor of calpains significantly reduced the damage [92]. Consistent with the function of calpains in cytoskeletal modulation, calpains were shown to contribute to the disruption of retinal endothelial cell cytoskeleton, leading to abnormally structured and dysfunctional neovessels, which is a proposed mechanism of hypoxia damage [93,94]. In addition to their elevated expression in the neural retina in glaucoma models, calpain levels were also increased in lens epithelial cells of cataract patients with diabetic retinopathy [95].

Cathepsins are another family of proteins functioning in programmed cell death, which are mainly cysteine proteases that degrade cellular and extracellular proteins under acidic environments [96,97]. The expression of cathepsins (Cathepsin A, B, D, E and S) is detected not only in RPE, where photoreceptor disks are degraded by phagocytosis and lysosomal degradation, but also in various other locations in the eye, including choroid, optic nerve and cornea. Additionally, cathepsin expression level is associated with the pathology of a variety of eye diseases [96]. A proposed molecular mechanism of keratoconus is the elevated levels of cathepsin B and G in abnormal keratocytes, which results in the destruction of both Bowman’s layer and the stroma, causing localized nerve thickening and leading to the progression of disease [98,99,100]. Cathepsin B, D, E, L and S are of great importance in maintaining photoreceptor function and their expression levels have been shown to increase in response to conditions of oxidative stress and aging [101,102,103,104]. Due to the critical roles of cathepsins in keratocytes and photoreceptor homeostasis, the activity of cathepsin is closely related to the pathogenesis of cornea and retina degenerative diseases.

Although the pathogenesis of eye diseases has been connected to the altered levels of calpains, cathepsins and the activated form of caspases, their expression is not equivalent to their protease activities when taking the presence of endogenous cysteine protease inhibitors, such as cystatin C (CysC), into consideration. The ratio between cysteine proteases and CysC is expected to be critical to the overall activity of cysteine proteases. Interestingly, a polymorphism in CysC (rs1064039, G>A) has been identified previously to associate with AMD [105], potentially through alteration of its signal sequence cleavage [106]. Due to the presence of endogenous inhibitors which act as an extra factor regulating activity, ABPP would be effective to directly evaluate cysteine protease activities instead of measuring just expression levels.

### 3.3. Matrix Metalloproteinases

Matrix metalloproteinases (MMPs) are zinc-dependent endopeptidases that degrade extracellular matrix (ECM) proteins and cell surface molecules, both of which are important in regulating extracellular tissue signaling networks [107,108]. MMPs are produced as zymogens and activated upon cleavage by proteases. Once cleaved, the activities of MMPs are often then controlled by endogenous inhibitors, such as tissue inhibitors of metalloproteinases (TIMPs) [109]. Since ECM maintenance and remodeling is important for ocular homeostasis, it is no surprise that MMPs/TIMPs have been correlated with multiple eye diseases [110]. Similar to serine proteases and cysteine proteases, altered expression levels and polymorphisms in MMPs have been detected in AMD, diabetic retinopathy and glaucoma. Specifically, MMP-1, 2, 9 and 14 have been shown to play a significant role in both early and advanced AMD. Altered expression of activated forms of MMPs and/or an imbalanced MMPs/TIMPs ratio in RPE or Bruch’s membrane choroid result in the accumulation of ECM structure components (e.g., drusen), compromised RPE function and the development of CNV [111,112]. In addition to increased ocular levels, the plasma level of MMP-9 was reported to also be increased in age-related maculopathy and CNV patients as well [113]. Furthermore, the risk of wet AMD has been suggested to correlate to the longer microsatellites in the MMP-9 promoter region [114]. However, a synonymous coding variant of MMP-2 (rs2287074 G>A) was reported to be a protective factor in AMD with unclear functional implications [115].

Aside from AMD, the participation of MMPs in the pathology associated with diabetic eyes has also been investigated. For example, levels of MMP-2 and MMP-9 were significantly higher in the retina and vitreous of diabetic retinopathy patients and animal models [116,117,118]. Yet, the molecular mechanism of how MMPs contribute to the disease is not clear. In the early stage of diabetic retinopathy, increased levels of MMP-2 and MMP-9 may promote vascular permeability by degrading tight junction proteins, thus disturbing the tight junction complex. This observation was confirmed by decreased transepithelial electrical resistance of RPE and retinal endothelial cell lines after the cells were treated with excess MMP-2 and MMP-9 [118]. Yet, another study indicated the role of MMP-2 in activating retina capillary cells apoptosis and enhancing cellular permeability by mitochondrial dysfunction [119]. In contrast to their involvement in early stages of diabetic retinopathy, increased levels of MMP-2 and MMP-9 facilitate angiogenesis in the later stage of the disease through multiple proposed mechanisms [116]. 

Moreover, glaucoma is another eye disease in which MMPs are implicated, most likely through mediating aqueous humor flow, which is achieved by remodeling of trabecular meshwork ECM, thus stabilizing outflow resistance and intraocular pressure [120]. Altered levels and activities of MMPs have been detected in the aqueous humor and tears of glaucoma patients, but the changes are not consistent among different MMPs and various types of glaucoma. For example, MMP-2 and MMP-9 levels were reduced in pseudoexfoliation glaucoma, while MMP-9 activity in tears was increased in primary open-angle and angle closure glaucoma [121,122]. Whereas MMP activities are typically assessed by zymography, this approach will measure activity of all MMPs present in a sample (enzymes, pro-enzymes, and even enzymes that were bound to TIMPs originally). Thus, at least in theory, ABPP would provide more comprehensive information of the ultimate activity each enzyme in its native state. The impact of MMPs on glaucoma pathogenesis has been correlated to SNPs. For example, the R279Q variant of MMP-9 (rs17576 G>A) was reported to be protective against the development of glaucoma in a Caucasian population, and MMP-9 rs3918249 C/C instead of C/T genotype in the promoter region was more frequent in glaucoma and AMD patients [123,124]. The functional impact of these SNPs needs further elucidation, which can be effectively achieved by ABPP.

### 3.4. Other Proteases

Additional other diverse types of proteases are also involved in ocular physiology and pathophysiology. In this section, we briefly introduce the function of β-secretase, an aspartic acid protease, and the ubiquitin-proteasome pathway (UPP, the catalytic subunit of the proteasome is a threonine protease) in the eye. It is well known that β-secretase is responsible for generating amyloid beta protein in the brain, the pathological hallmark of Alzheimer’s disease [125,126]. Recently, the involvement of β-secretase in retinal homeostasis has also been recognized in the course of the development of a novel β-secretase inhibitor [127,128]. The inhibition of β-secretase activity causes retinal dysfunction, including lipofuscin accumulation, retinal thinning and accelerating CNV in mice and rat [127,128]. The elevation of β-secretase expression was detected in response to stress conditions, such as oxidative stress, a risk factor of AMD [127]. The UPP is significantly important in many cellular functions, including protein quality control and signal transduction. In the eye, the degradation of unfolded and damaged proteins by the UPP is critical for cellular waste clearing and maintaining eye functions [129]. Therefore, the UPP is associated with many eye diseases, in which the etiology is related to the overburden of damaged proteins in eye tissues, such as retinal degeneration, cataract [130] and some types of glaucoma [131].

## 4. Application of ABPP in Eye Research

In the previous section, we summarized a number of representative proteases that are relevant to eye diseases. Previous ‘omics’-centered studies focused primarily on genome-wide association analysis and detection of protease expression at transcription and protein levels, neither of which directly probes protease activities. Such studies do not take into consideration the binding of endogenous inhibitors which is an important regulatory process controlling protease activities such as the scenario with MMPs/TIMPs [109]. Therefore, studies focusing on protease levels, or even levels of activated protease, are not appropriate reflections of ultimate enzyme activity. Instead, ABPP only probes the active proteases, thus providing an assessment of the protease activity, despite being different from conventional enzymology. Moreover, the pathogenesis of eye diseases is complex, and each disease has been shown to be influenced by multiple families of proteases. However, most follow-up, in-depth studies only investigate the function of a single protease in disease development, thus overlooking the cooperation and competition of other proteases that are also associated with the same disease, and fail to obtain a comprehensive understanding of eye diseases. Due to the failure of previous studies to address the above questions, it is necessary to profile a broad range of proteases and link the activities of proteases directly to pathology, which can be achieved by the application of ABPP to eye research. At last, we propose different types of experiments that can be performed with ABPP to study eye diseases.

On one hand, ABPP can be utilized to compare protease activities between normal and disease conditions for a mechanistic study, for biomarker discovery, and diagnosis. Traditional proteomics can profile global expression of more than 1000 proteins including a significant number of proteases in different locations of the eye, such as tears, lacrimal fluids, vitreous humor, lens, retina, and characterize the differences between the healthy population and patients with eye diseases, such as dry eye, AMD, diabetic retinopathy, cataract and vitreoretinal disease [132,133,134,135,136,137,138,139]. Rather than protein abundance, the utilization of ABPP can obtain an activity mapping of proteases and identify the most critical and active players in eye diseases. The change in protein abundance indirectly indicates the correlation to diseases, but the activity of the protein directly influences disease pathogenesis, which helps to reveal the potential mechanisms of the disease and provide biomarkers for diagnosis and prognosis. For example, a strategy has been developed to enrich and identify proteases in the human trabecular meshwork, including serine and cysteine proteases [140]. Tripeptidyl peptidase II was identified in this study, which indicated impaired proteasome function [140]. ABPP can also be performed on the retina and RPE proteome to evaluate the respective contributions of different categories of proteases to AMD development, thus obtaining a better understanding of the molecular mechanism of AMD etiology. Moreover, due to the accessibility and ease of sample collection of tear fluids, or even vitreous samples, the activities of proteases in patient fluids can also be assessed and correlated with various eye diseases, such as dry eye syndrome, Sjogren syndrome, complications due to diabetes, conjunctivitis and others. Once found, the biomarker can then be used for diagnosis as well.

**Figure 3:**
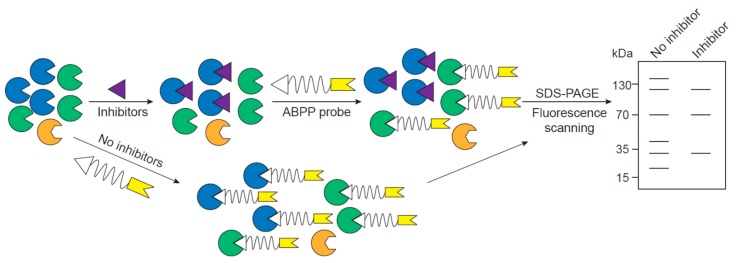
Experimental scheme to identify protease inhibitors and their target enzymes using ABPP. An ABPP competition experiment involves addition of potential inhibitors of interest followed by labeling remaining enzymes with an ABPP. Through a comparison of a sample treated with only probe, one can identify specificity and targets of potential protease inhibitors.

On the other hand, after the proteases that relate to diseases are identified, ABPP can also be used for inhibitor/activator discovery and drug development to provide treatment for the disease by targeting specific, or a group of, proteases. This experiment would be performed in competitive mode, where the proteome is incubated with inhibitors/activators first and then labeled with ABPP probes for final detection (Figure 3) [30]. Since inhibitor binding blocks the active site of protease, which prevents the labeling by an ABPP probe, effective inhibitors cause the disappearance of their corresponding proteins targets in the final detection when compared to ABPP probing without inhibitor incubation (Figure 3). This method has many advantages over the traditional inhibitor screening, for example, probing the whole proteome avoids the effort of making recombinant proteins, knowing the substrate of each protease is not necessary, and the selectivity of the inhibitor to a broad range of proteases can be evaluated simultaneously. For example, the Corson and Seo groups synthesized a novel antiangiogenic homoisoflavonoid derivative, SH-11037, and showed that this compound can prevent angiogenesis in CNV models both in vitro and in vivo [141,142]. To characterize the mechanism of this compound and find the target of the inhibitor, they performed ABPP with a SH-11037-based probe and identified soluble epoxide hydrolase, a key enzyme for epoxy fatty acid metabolism, as its binding partner, inhibition target, and main contributor to CNV disease [143].

Overall, the potency of ABPP lies in the specificity of the probe to target a group of proteases with similar activity. Probes for the most representative types of the aforementioned proteases, such as serine proteases, cysteine proteases and matrix metalloproteinases, have been developed and applied in studies. In some other situations where particular proteases or subfamilies need to be profiled and specific ABPP probes are not available, effort needs be invested into the design of the corresponding probes before their use in ocular research.

## 5. Conclusions

ABPP is a powerful technique to probe protease activities that have not been investigated directly or comprehensively in eye diseases. Polymorphisms/mutations in various proteases, and/or altered expression is inexorably linked to the pathogenesis of eye diseases. However, the connection between how alterations in proteases ultimately affects their activity in complex systems, and how this relates to pathology needs to be solidified. ABPP provides the feasibility to study the importance of protease activities in this context and enables the identification of potential targets for therapeutic treatment of eye diseases.
